# Correspondence

**Published:** 1914-05

**Authors:** George H. Norton

**Affiliations:** Deputy Engineer Commissioner


### CORRESPONDENCE

This department is intended for the presentation of news and views not coming within the scope of other departments, and particularly to afford opportunity for discussion and criticism of views elsewhere expressed.

DEPARTMENT OF PUBLIC WORKS

Bureau of Engineering.

Buffalo, N. Y.

March 2d.

Dr. John D. Bonnar,

144 Jewett Avenue,

Buffalo, N. Y.

Dear Sir:—

I had the honor to appear before the Erie County Medical Society on February 16th, at my own suggestion, with expectation of opening a sharp discussion and fully succeeded, but with sore disappointment that in a full hour discussion, no attempt was made by any physician to meet the line of argument put forth.

The remarks made were not intended to be local or personal, but rather as from the engineer to the physician, to explain why the medical profession continues to talk so much about the one minor disease, typhoid, in which the engineer may be expected to lend material assistance, and so little about the more prevalent communicable disease, commonlv considered as beyond engineering assistance.

To be specific, after a season of unusual gales and roily water, January's vital statistics for Buffalo showed deaths: typhoid 3; diptheria 4; syphilis 6; whooping-cough 12; tuberculosis, pul., 52. Several physicians have seen fit to make severe strictures on water as producing typhoid—why has not the engineer an equal justification to ask, as to these other diseases?—and to ask why you cannot, with equal expenditure, save at least two lives, from these other diseases, to one from typhoid by water filtration?

I am fully aware that this was a novel view-point to many of you and you were not prepared to meet it, yet it is a definite issue, which is up to you to meet, not by generalities, but by sober and logical argument.

The engineer has been brought into the public health problem and must apply his principles to it. He may make unwarranted assumptions, through his limited knowledge of medicine but he will insist on being shown his errors, and the physician should be open to meet these arguments, on their merits, and not assume to ignore them.

I await your answer.

Yours very truly,
GEORGE H. NORTON,
Deputy Engineer Commissioner.

---

March 23d, 1914.

George H. Norton, Esq.,

Deputy Engineer Commissioner,

Buffalo, N. Y.

Dear Sir:—

